# The influence of parental smoking and family type on saliva cotinine in UK ethnic minority children: a cross sectional study

**DOI:** 10.1186/1471-2458-10-262

**Published:** 2010-05-20

**Authors:** Melissa J Whitrow, Seeromanie Harding, Maria J Maynard

**Affiliations:** 1 Social and Public Health Sciences Unit, Medical Research Council, Glasgow, UK

## Abstract

**Background:**

In the United Kingdom, there has been an increase in cigarette smoking in ethnic minority adults since the 1970s; in some groups levels are now similar to that of White British people. We aimed to examine the determinants of exposure to secondhand smoke in ethnic minority children. We hypothesised that exposure to secondhand smoke in children will vary across ethnic groups, but that the correlates of exposure would be similar to that of Whites.

**Methods:**

The Determinants of Adolescent Social well-being and Health sample comprises 3468 White United Kingdom and ethnic minority (Black Caribbean, Black African, Indian, Pakistani, Bangladeshi) pupils aged 11-13 yrs. Outcome was saliva cotinine concentration. Explanatory variables collected by self-complete questionnaire included ethnicity, child reported household smoking and socio-economic circumstances. Data were analysed using linear regression models with a random intercept function.

**Results:**

Ethnic minority children had lower saliva cotinine than Whites, partly explained by less smoking among parents. White and Black Caribbean children had higher cotinine levels if they lived in a household with a maternal smoker only, than with a paternal smoker only. Living in a lone compared to a dual parent household was associated with increased cotinine concentration of 45% (95%CI 5, 99%) in Whites, 27% (95%CI 5,53%) in Black Caribbeans and 21% (95%CI 1, 45%) in Black Africans after adjusting for household smoking status. Material disadvantage was a significant correlate only for White children (40% (95%CI 1, 94%) increase in cotinine in least compared to most advantaged group).

**Conclusions:**

Ethnic minority children were less exposed to secondhand smoke than Whites, but the variations within groups were similarly patterned. These findings suggest that it is important not to be complacent about low smoking prevalence in some minority groups.

## Background

Exposure to the cigarette smoke of others during childhood has been linked to a range of adverse health outcomes including low birth weight, poor lung development, and increased risk of respiratory disorders such as asthma and bronchitis[1]. Parental tobacco smoking is the predominant source of passive smoking in children[2], and many studies rely on self reported parental smoking as an indication of passive smoke exposure in childhood. Previous studies [2,3] have determined the association between parental smoking and childhood passive smoking using biomarkers of exposure such as saliva or urine cotinine concentration, often finding maternal smoking is associated with higher cotinine concentration than paternal smoking[3]. We are not aware of any United Kingdom (UK) studies that have examined the determinants of exposure to secondhand smoke in ethnic minority children.

There is a need for such investigation given the rapid changes in smoking habits of ethnic minorities in the UK. In the late 1970s the prevalence of smoking in ethnic minorities was less than half that of the national prevalence (i.e. the standardized smoking ratio for Black Caribbeans was 46 and for South Asians 38) [4,5]. By 1992 this pattern had changed dramatically, with Black Caribbeans just as likely to smoke as their White peers[6]. In 2004, compared with White UK men, smoking prevalence remained similar in Black Caribbeans (24% and 25% respectively)[7]. However, by 2004 there were differences in the prevalence amongst the South Asian men. Smoking prevalence was lower in Indians (20%), but higher in Pakistani (29%) and Bangladeshi (40%) men compared to the White UK group [7]. In 2004, Black Caribbean women had similar smoking prevalence to White UK women (24% and 23% respectively)[7], whereas Black African (10%) and South Asian (i.e. Bangladeshis 2%) women had lower rates. Gender differences in tobacco smoking is culturally patterned in Pakistanis and Bangladeshis [8,9]. It is also possible cultural taboos lead to under-reporting of smoking. Bangladeshi men and women have been found to have lower self-reported smoking rates than suggested by their salivary cotinine concentration[7].

We are aware of only one study that has used an objective measure of passive smoking exposure (such as cotinine) to examine the exposure of ethnic minority children to passive smoking in the UK. Black and Asian children aged 4-15 years in the Health Survey for England in 2004 had lower salivary cotinine concentrations than their White peers[10]. The ethnic groups were aggregated due to small sample sizes and correlates were not investigated.

Given ethnic differences in smoking habits in adulthood and in the cultural milieu that patterns smoking conduct, we would expect differences in exposures to secondhand smoke in childhood. We used data from the Determinants of Adolescent Social well-being and Health (DASH) study, which contains a large number of ethnic minority children, to examine exposure to secondhand smoke using salivary cotinine among White British, Black Caribbean, Black African, Indian, Pakistani and Bangladeshi children aged 11-13 years. We hypothesised that exposure to secondhand smoke in children will vary across ethnic groups, but that the correlates of exposures would be similar to that of Whites.

## Methods

### Study sample and design

The DASH study has been described previously[11]. Briefly, the sample was recruited from 51 schools in ten inner London boroughs with high proportions of the main ethnic minority groups. All pupils from Years 7 and 8 (aged 11-13 years) in randomly selected mixed ability classes were invited to join the study and took part in their schools in 2003-04 (prior to implementation of the workplace and public place smoking ban in England). Children completed a structured questionnaire under the supervision of trained fieldworkers. The questionnaire is available at http://www.sphsu.mrc.ac.uk/study-sites/dash/. It collected self reported information on demographic, socio-economic circumstances, family life and health, with specific aim of examining the social patterning of risk within ethnic groups. Approvals from the Multi-centre Research Ethics Committee and from Local Education Authorities were obtained. Active (opt-in) consent was used for pupils and passive (opt-out) consent for parents. The pupil response rate was 81%.

Ethnicity of White UK (this category did not include White children from outside the UK), Black Caribbean, Black African, Indian, Pakistani and Bangladeshi origin was self defined. Pupils who reported 'Black British' or 'Asian British' or who did not report their own ethnicity were classified using reported parental ethnicity and parental and grandparental country of birth using a rule of having at least one parent with an ethnicity reflecting home countries of grandparents and having at least three grandparents who were born in the home countries A random sample of children who reported their ethnicity as White UK (n = 515 taken from original sample of 1236), and all of those who reported Black Caribbean (n = 875), Black African (n = 1017), Indian (n = 462), Pakistani (n = 385) or Bangladeshi (n = 217) ethnicity were selected to have a saliva sample assayed for cotinine. Children from other ethnic groups were excluded from this study. Two schools declined to take part in the saliva sample component of the study (n = 105). Of the selected children, 2411 (70% of sample) had sufficient saliva in their sample for the assay[12] and completed questions on their smoking behaviour (75% of the White UK sample, 67% Black Caribbeans, 64% Black Africans, 75% Indians, 73% Pakistanis and 76% Bangladeshis). Black Caribbeans (n = 144, OR 2.5, 95%CI 1.7-3.6) and Black Africans (n = 186, 2.8, 1.9-4.0) were more likely to have incomplete smoking data than White UK (n = 38). Children who were self reported smokers (I smoke regularly (one or more cigarettes a week)", or "I smoke occasionally (sometimes)"), who reported smoking one or more cigarettes in the previous week, or were biochemically confirmed smokers (salivary cotinine concentration > 15 ng\mL[13]) were excluded from the analysis (n = 100, 3% of sample).

### Outcome and exposure measures

Children were told prior to the study that a saliva sample would be taken from them for the purpose of detecting cotinine. Cotinine is a widely accepted biomarker of exposure to tobacco smoke and has a half life of up to 24 hrs[1]. Samples were taken directly from school on the day of collection by black bag to freezers (-20 degrees). After all samples were collected, they were transported frozen to labs for analysis. Salivary cotinine was assayed by capillary Gas Chromatography with a detection limit of 0.1 ng/mL[12].

Household smoking was measured by two questions; "Do any of the people you live with smoke tobacco (cigarettes, roll-ups, cigars or a pipe)?" (asked for mother, step-mother, father, step-father, brother or sister, and someone else you live with), and "about your parents (you live with), do they smoke cigarettes?" (asked for mother and father). These questions were cross-checked with a question on whom the child lived with. Exposure to passive cigarette smoke was affirmed if the child resided with one or more people who smoke cigarettes. This was further classified by the person who smoked (mother/step-mother, father/stepfather, both parents or other).

Measuring socio-economic circumstances among minority groups is complex, and a multi dimensional index appears to be more discriminating of health differences [14,15]. It is also problematic to obtain some information (e.g parental occupation) from children. With this in mind, socio-economic circumstances were measured by the sum of 17 standard of living items (variable referred to as material disadvantage, items were family vehicle, CD player/hi fi system, television/DVD player, garage, bedrooms, computer, toilet, holiday abroad each year, deep freeze or fridge freezer, dishwasher, garden, washing machine, microwave oven, satellite/cable/digital TV, tumble dryer), family type (lone parent versus dual parent household) and crowding (more than one resident per bedroom).

### Model building

Cotinine data were positively skewed, hence the data were transformed by natural logarithm. Data were analysed using linear regression models with a random intercept function to adjust the analyses for clustering within schools. All models included the natural logarithm of cotinine as the dependent variable and were initially adjusted for sex, age (continuous) and the day of the week the sample was taken (Monday versus other week days, assuming that likelihood of exposure is greater on Monday due to longer exposure times at the weekend). This is referred to as the partially adjusted model. To examine other contributors to ethnic differences in salivary cotinine, household smoking and socioeconomic circumstances variables were added to the partially adjusted model with ethnicity as the main explanatory variable. There were significant (p < 0.05) interactions between ethnicity and household smoking status, and ethnicity and disadvantage. To identify independent correlates of cotinine for each ethnic group we ran ethnic specific multivariable models that included sex, age, day of the week, household smoking status and the socioeconomic circumstances variables. Data presented are back transformed (exponentiated) adjusted geometric means and 95% confidence intervals (CI), or relative change, that is the percentage difference (and 95%CI) from the reference group (calculated as the exponential function of the coefficient minus one, times 100%). Differences between groups were considered significant if p < 0.05.

## Results

Tables 1 and 2 show the percentage distributions of children and also partially adjusted (age, sex, day of week) geometric mean cotinine concentrations by household smoking status and socioeconomic circumstances variables. Ethnic minority children were less likely than White UK children to live with a mother who smoked cigarettes or with two parents who smoked. Bangladeshi children were more likely and Black Africans less likely to live with a paternal smoker than White UK. With the exception of Indian children, ethnic minorities were more likely to be in the least advantaged tertile of material disadvantage compared to their White UK peers.

**Table 1:** Child reported household smoking and socioeconomic circumstances for each ethnic group.

	White UK (N = 359)	Black Caribbean (N = 551)	Black African (N = 633)	Indian (N = 339)	Pakistani (N = 275)	Bangladeshi (N = 154)
	% (95%CI)	% (95%CI)	% (95%CI)	% (95%CI)	% (95%CI)	% (95%CI)
Age - mean (SE)	12.6 (0.03)	12.7 (0.03)	12.7 (0.03)	12.5 (0.03)	12.6 (0.04)	12.7 (0.05)

Females	47 (42, 52)	52 (48, 56)	54 (50, 58)	44 (39, 49)	32 (27, 38)	38 (31, 46)

*Household smoking*						
No smokers	43 (38, 48)	47 (43, 51)	77 (74, 80)	73 (68, 78)	60 (54, 66)	49 (41, 57)
Maternal smoker only	19 (15, 24)	15 (12, 18)*	3 (2, 4)*	2 (1, 4)*	1 (0, 3)*	4 (2, 8)*
Paternal smoking only	13 (10, 17)	11 (9, 14)	8 (6, 10)*	14 (11, 19)	20 (16, 26)	32 (26, 40)^||^
Both parents smoke	14 (11, 18)	6 (5, 9)*	1 (1, 3)*	2 (1, 4)*	3 (1, 6)*	1 (0, 5)*
Other household member smokes	4 (3, 7)	8 (6, 11)	3 (2, 5)*	4 (2, 6)*	8 (5, 12)	6 (4,12)

*Socioeconomic circumstances*						
Most advantaged tertile°	47 (42, 52)	30 (26, 34)	21 (18, 25)	37 (32, 42)	28 (23, 34)	16 (11, 22)
Least advantaged tertile°	19 (15, 23)	25 (22, 29)^||^	31 (28, 35)^||^	16 (13, 21)	24 (19, 29)^||^	32 (25, 40)^||^
Dual parent household	78 (73, 82)	56 (52, 60)	64 (60, 67)	94 (90, 96)	88 (84, 92)	86 (79, 90)
Lone parent household	20 (16, 25)	38 (35, 43)^||^	29 (26, 33)^||^	5 (3, 8)*	11 (7, 15)*	12 (8, 19)*
Not crowded household	52 (46, 57)	40 (36, 44)	21 (18, 24)	34 (29, 39)	17 (13, 22)	16 (11, 22)
Crowded household	41 (36, 46)	50 (46, 54)^||^	67 (63, 70)^||^	50 (45, 55)^||^	73 (67, 78)^||^	75 (68, 82)^||^

° Tertiles of 17 standard of living items; * Significantly (P < 0.05) lower than corresponding value for White UK, ^|| ^significantly (P < 0.05) higher than corresponding value for White UK.

**Table 2:** Partially adjusted^† ^salivary cotinine concentration (geometric means) for each ethnic group.

	White UK	Black Caribbean	Black African	Indian	Pakistani	Bangladeshi
	**Cotinine ng/mL (95% CI)**^†^	**Cotinine ng/mL (95% CI)**^†^	**Cotinine ng/mL (95% CI)**^†^	**Cotinine ng/mL (95% CI)**^†^	**Cotinine ng/mL (95% CI)**^†^	**Cotinine ng/mL (95% CI)**^†^
*All*	0.71 (0.62, 0.82)	0.50 (0.44, 0.56)*	0.29 (0.26, 0.33)*	0.27 (0.23, 0.31)*	0.32 (0.27, 0.37)*	0.50 (0.41, 0.60)*

*Household smoking*						
No smokers	0.30 (0.25, 0.36)	0.32 (0.28, 0.36)	0.26 (0.23, 0.29)	0.22 (0.19, 0.26)*	0.25 (0.21, 0.29)	0.32 (0.26, 0.41)
Maternal smoker only	1.48 (1.16, 1.89)	1.08 (0.86, 1.35)*	0.65 (0.39, 1.07)*	0.53 (0.23, 1.23)*	0.94 (0.22, 3.94)	0.72 (0.32, 1.64)
Paternal smoking only	0.84 (0.63, 1.11)	0.44 (0.34, 0.56)*	0.49 (0.38, 0.64)*	0.46 (0.35, 0.61)*	0.49 (0.38, 0.65)*	0.88 (0.67, 1.16)
Both parents smoke	1.97 (1.44, 2.69)	1.43 (0.99, 2.05)	0.46 (0.22, 0.94)*	1.23 (0.51, 2.97)	0.41 (0.19, 0.87)*	0.33 (0.04, 2.89)
Other household member smokes	0.61 (0.36, 1.05)	0.43 (0.32, 0.60)	0.44 (0.27, 0.71)	0.33 (0.18, 0.61)	0.35 (0.22, 0.55)	0.35 (0.19, 0.63)

*Socioeconomic circumstances*						
Most advantaged tertile°	0.57 (0.46, 0.70)	0.45 (0.37, 0.54)	0.31 (0.25, 0.38)*	0.28 (0.22, 0.36)*	0.34 (0.26, 0.45)*	0.57 (0.36, 0.90)
Least advantaged tertile°	1.09 (0.84, 1.43)	0.51 (0.42, 0.61)*	0.29 (0.25, 0.35)*	0.27 (0.20, 0.37)*	0.27 (0.21, 0.36)*	0.47 (0.34, 0.64)*
Dual parent household	0.65 (0.56, 0.76)	0.46 (0.40, 0.53)*	0.28 (0.25, 0.32)*	0.27 (0.24, 0.32)*	0.31 (0.26, 0.36)*	0.50 (0.41, 0.61)*
Lone parent household	0.96 (0.74, 1.26)	0.53 (0.45, 0.62)*	0.32 (0.27, 0.37)*	0.26 (0.15, 0.46)*	0.38 (0.25, 0.58)*	0.45 (0.27, 0.75)*
Not crowded household	0.53 (0.44, 0.63)	0.47 (0.40, 0.55)	0.29 (0.24, 0.36)*	0.27 (0.22, 0.34)*	0.30 (0.22, 0.42)*	0.41 (0.26, 0.64)
Crowded household	0.89 (0.73, 1.07)	0.51 (0.44, 0.59)*	0.29 (0.25, 0.33)*	0.28 (0.24, 0.34)*	0.31 (0.26, 0.37)*	0.50 (0.41, 0.62)*

^† ^Cotinine adjusted for age, sex and day of week (Monday vs other); ° Tertiles of 17 standard of living items; * Significantly (P < 0.05) lower than corresponding value for White UK.

Overall levels of cotinine were lower in ethnic minority children than White UK (Table 2). Indian children in non-smoking households had lower cotinine than White UK children in non-smoking households. Black Caribbeans, Black Africans, Indians and Pakistanis generally had lower cotinine within each household smoking category compared to White UK children in the corresponding category. Ethnic minorities had generally lower cotinine compared to White UK in the same categories of the socioeconomic circumstances variables.

Adjusting for ethnic differences in household smoking status reduced the ethnic differences in salivary cotinine for all groups (difference from White UK (95%CI): Black Caribbeans -20% (-30, -8%); Black Africans -35% (-44, -25%); Indians -44% (-52, -34%); Pakistani -37% (-47, -25%)) and removed the cotinine advantage of the Bangladeshis compared with the Whites (-9% (-25, 11%)). Adjustment for socioeconomic circumstances had no influence on ethnic differences in cotinine (data not shown).

Household smoking status was an independent correlate of cotinine within every ethnic group (Table 3). White UK and Black Caribbean children had higher cotinine levels if they lived in households with a maternal smoker only than with a paternal smoker only. Family type was a significant correlate for some ethnic groups, residing in a lone compared to a dual parent household was associated with increased cotinine concentration of 45% (95%CI 5, 99%) in White UK, 27% (5, 53%) in Black Caribbeans and 21% (1, 45%) in Black Africans after adjusting for household smoking status. Material disadvantage was a significant correlate only for White UK children, being in the least advantaged tertile associated with a 40% (1, 94%) increase in cotinine compared to those in the most advantaged tertile.

**Table 3:** The influence of household smoking status on salivary cotinine concentration within each ethnic group, fully adjusted model^†^

	No smokers in household	Maternal smoker only	Paternal smoker only	Both parents smoke	Other household member smokes
*Salivary cotinine*	ng/mL (95%CI)	% difference from non smoking households within group (95%CI)			
		
White UK	0.35 (0.28, 0.43)	338.0 (214.9, 509.2)*	148.2 (71.7, 258.8)*	520.4 (330.7, 793.8)*	53.3 (-13.1, 170.2)
Black Caribbean	0.30 (0.26, 0.35)	228.3 (157.8, 318.0)*	54.7 (17.0, 104.5)*	417.3 (266.1, 630.9)*	27.8 (-6.3, 74.5)
Black African	0.26 (0.23, 0.29)	136.9 (46.6, 282.8)*	108.7 (58.1, 175.4)*	104.2 (9.0, 282.6)*	50.7 (-3.0, 134.1)
Indian	0.23 (0.19, 0.27)	161.7 (19.3, 474.3)*	109.3 (58.3, 176.9)*	453.6 (159.8, 1079.5)*	64.0 (-5.5, 184.5)
Pakistani	0.26 (0.22, 0.31)	212.5 (-12.4, 1015.7)	108.5 (57.8, 175.6)*	55.0 (-19.3, 197.9)	56.5 (3.5, 136.6)
Bangladeshi	0.28 (0.22, 0.34)	177.5 (32.9, 479.4)*	230.0 (136.3, 360.8)*	34.8 (-75.8, 649.8)	43.8 (-17.5, 150.6)

* significantly different from non-smoking households P < 0.05; ^† ^adjusted for age, sex, day of week, material disadvantage, family type and crowding

## Discussion

Contrary to our hypothesis, all non-smoking children in minority groups had significantly lower salivary cotinine concentration than White UK children. This was the case even for groups (such as Black Caribbeans) where women have been reported to have similar levels of smoking to White women in adulthood. Less smoking among parents accounted for a large part of this advantage. In fully adjusted models, Black Caribbean, Black African and White UK children in lone parent households had higher cotinine levels than those in dual parent households. This difference remained after adjusting for household smoking and material disadvantage.

South Asian children have been reported to have lower saliva cotinine concentration even after adjustment for parental and own smoking[16]. We found no significant difference in cotinine between Bangladeshi and White children after adjustment for parental smoking in non-smoking children. The likely reason for this is the heterogeneity in smoking habits within the South Asian group, for example smoking is more socially acceptable amongst Bangladeshi than Pakistani men[17].

Black Caribbean, Black African, Indian and Pakistani children had lower cotinine than White UK children after adjustment for cigarette smoking of parents. This is likely to be due to ethnic differences in the amount of cigarettes smoked by parents, and/or the frequency of smoking in front of their children. Ethnic minority groups in the UK are less likely to smoke 20 or more cigarettes a day than White UK men and women[7]. Cigarette smoking is culturally unacceptable in some groups, particularly in women [8,9]. This may result in parents refraining from smoking in front of their children, which would reduce exposure to secondhand smoke.

Cigarette smoking is socially patterned in the general population; those earning low incomes or unemployed are more likely to be smokers, as are lone parents[18]. After adjustment for household smoking status, material disadvantage was an independent correlate of exposure to secondhand smoke only in White UK children. This suggests the quantity of cigarettes smoked by parents or the likelihood of smoking in the presence of children is socially patterned in this group. The Health Survey for England (HSE) showed smoking was associated with income in the general white population and in Black Caribbean women, but not in other ethnic minority groups[7]. Exposure to secondhand smoke may not be socio-economically patterned in ethnic minority groups due to alternative influences on smoking such as religion and cultural traditions [8,9].

In the White UK and Black Caribbean group maternal smoking was more strongly associated with secondhand smoke than paternal smoking in DASH. This difference may be due to the amount of time mothers spend with their children compared to fathers[3]. White UK, Black Caribbean and Black African children in lone parent households had a 21-45% increase in cotinine concentration compared to their dual parent household peers. Disadvantage[19] and parental responsibility is greater in lone parents, potentially driving a higher number of cigarettes smoked, and increasing the likelihood of smoking around children. This is the first study to report this lone parent effect in Black Caribbean and Black African groups.

Our study is subject to limitations. Cotinine is an objective measure of short term (18-20 hours) exposure to tobacco smoke[1] so we are unable to draw conclusions on long term exposure to tobacco smoke. However adjustment for the day of the week that the saliva sample was taken enabled us to allow for differences in household smoking exposure on weekdays compared with weekends. African American children tend to have greater cotinine concentration than Whites after adjustment for detailed measures of exposure to smoke[20]. The metabolism and clearance of cotinine varies between individuals and may vary by ethnicity, possibly due to polymorphisms in the *CYP2A6 *gene which encodes the cytochrome P450 (CYP) 2A6 responsible for the metabolism of nicotine to cotinine[21]. However more recent literature suggests no ethnic difference in nicotine metabolism[22]. As we found lower cotinine concentration in ethnic minority children, the potential overestimation of smoke exposure in minorities due to slower cotinine metabolism would bias our results towards the null (an underestimation of the lower secondhand smoke exposure in ethnic minorities compared to Whites). It is possible that some of the 30% of children who did not provide enough saliva did so intentionally as they were told prior to the study that a saliva sample would be taken to detect cotinine. It is likely these children were smokers keen to hide their own smoking, hence they would have been excluded from the analyses regardless.

DASH was limited by the amount of information collected on family smoking habits and reliance on reports from children. It is also possible that children underreported family smoking habits due to awareness of their harmful effect. Collection of parental self reported smoking information (such as cigarettes smoked per day and proximity to the child while smoking) would have enabled more detailed conclusions on the reasons for ethnic differences in cotinine within each category of household smoking. It is likely children in the study were also exposed to secondhand smoke outside of the home, potentially explaining why some children from non-smoking homes had detectable levels of cotinine, however data on this was not collected. The percentage of children exposed to secondhand smoke within some ethnic groups and social strata were low (< 5%), reducing the reliability of cotinine concentration in these groups. Hence larger studies could be useful to confirm our results. Ethnicity by gender interactions were not significant, implying no difference in exposures, although we cannot rule out inadequate power to detect a difference. As with any school based study the DASH sample only included children who attended school on one of the data collection days. School truants and those absent due to illness or disciplinary measures may be under represented in the final sample[23].

## Conclusion

In conclusion, ethnic minority children had less exposure to secondhand smoke than Whites, with evidence for social patterning in the White UK, Black Caribbean and Black African children. Future research on exposure to secondhand smoke in children would benefit from collecting detailed ethnic specific information on the amount and location of parental smoking. These findings suggest that it is important not to be complacent about low smoking prevalence in some minority groups, and to be aware of heterogeneity within broad ethnic groups. Intervention programmes to support smoking reduction and cessation need to cover all groups.

## Competing interests

The authors declare that they have no competing interests.

## Authors' contributions

MW produced the first draft of the manuscript and conducted the analyses. MW, SH and MM formulated the research question and redrafted the paper. SH and MW are the guarantors of the paper. SH is the Principal investigator of DASH. All authors have seen and approved the final version of the paper.

## Funding

This work was supported by the Medical Research Council WBS code U.1300.00.003.00001.01. The research was conducted independently of the funder.

## Pre-publication history

The pre-publication history for this paper can be accessed here:

http://www.biomedcentral.com/1471-2458/10/262/prepub
